# Evaluating the prognostic relevance of neutrophil-to-lymphocyte ratio in cervical cancer: a systematic review and meta-analysis

**DOI:** 10.3389/fonc.2024.1461175

**Published:** 2024-12-23

**Authors:** Xieyan Zhuang, Yan Li, Hongfeng Zheng, Langjing Fu

**Affiliations:** Gynecology Department of Mingzhou Hospital, Ningbo, Zhejiang, China

**Keywords:** cervical cancer, neutrophil-lymphocyte ratio, survival, prognostic, meta-analysis

## Abstract

**Background:**

Recently, the neutrophil-to-lymphocyte ratio (NLR) has emerged as a promising prognostic marker for survival outcomes in individuals affected cervical cancer. However, research specifically focusing on the prognostic relevance of NLR across different cancer stages and in cases of recurrent metastases remains scant.

**Methods:**

We executed a systematic review of the literature from databases including PubMed, Embase, the Cochrane Library, and Web of Science, covering publications up to March 3, 2024. Studies evaluating the relationship between NLR and patient clinical outcomes were retrieved, guided by specifically defined inclusion and exclusion parameters. The key goals were to assess progression-free survival (PFS) and overall survival (OS), measured through hazard ratios (HR) and 95% confidence intervals (CI).

**Results:**

This meta-analysis encompassed 38 retrospective cohort studies, including data from 10,246 patients. The aggregated data showed that patients with increased NLR prior to treatment exhibit reduced OS (HR = 1.58, 95% CI: 1.44-1.74; p < 0.00001) and decreased PFS (HR = 1.48, 95% CI: 1.34-1.63; p < 0.00001). Furthermore, elevated NLR significantly impacted disease-free survival (HR: 1.79, 95% CI: 1.18-2.71; p = 0.006), recurrence rates (HR: 2.18, 95% CI: 1.36-3.51; p = 0.001), recurrence-free survival (HR: 3.05, 95% CI: 1.79-5.19; p < 0.0001), and the incidence of distant metastases (HR: 1.73, 95% CI: 1.20-2.50; p = 0.003).

**Conclusion:**

An elevated NLR prior to treatment demonstrates a strong association with decreased OS and PFS among patients with cervical cancer, underscoring the significance of NLR as a prognostic marker within this population.

**Systematic review registration:**

https://www.crd.york.ac.uk/PROSPERO/display_record.php?RecordID=529817, identifier CRD42024529817.

## 1 Introduction

Globally, cervical cancer holds the fourth position in the list of cancers most prevalent in women, in terms of disease incidence and fatality rate. As per 2022 information, roughly 660,000 fresh diagnoses and 350,000 deaths were documented (1). The primary modalities for managing cervical cancer involve surgical intervention or radiation therapy, with chemotherapy serving as an important adjunctive treatment. In early-stage detection, surgical intervention proves most effective. However, for individuals affected with locally advanced cervical cancer, the optimal approach is concurrent chemoradiotherapy (CCRT), which aids in curtailing both local and systemic recurrences (2). Unfortunately, in numerous underdeveloped countries, a majority of cervical cancer cases (> two-thirds) only come to light at more advanced stages. Individuals with locally advanced disease exhibit poorer survival rates and higher recurrence compared to those diagnosed at early stages, with five-year survival rates post-optimal treatments like chemoradiotherapy ranging between 31% and 55% (3). Traditional clinical factors such as tumor size and parametrial involvement significantly influence prognosis and are key components of the International Federation of Gynecology and Obstetrics (FIGO) staging system. Nevertheless, these conventional pathological makers fall short in both identifying the most effective treatment regimens and predicting clinical outcomes. Notably, patients with analogous pathological features and similar clinical tumor stages often experience diverse prognoses, highlighting the challenge in forecasting outcomes for cervical cancer. Thus, exploring novel clinical and prognostic markers is urgent (4).

Extensive research has demonstrated the critical influence of the tumor microenvironment, particularly inflammation, in cancer development, progression, metastasis, and prognosis (5–7). Inflammatory responses in patients are generally associated with poorer treatment outcomes and survival rates (7). Emerging studies supported the theory that inflammation contributes to the genesis and advancement of various solid and gynecological tumors (8). The prognostic significance of inflammatory biomarkers, such as neutrophil–lymphocyte ratio (NLR), monocyte-lymphocyte ratio (MLR), and platelet-lymphocyte ratio (PLR), has been thoroughly investigated in cervical cancer-related studies (9). Meta-analysis data by Zou et al. (10), involving 6,041 cervical cancer patients, pinpointed a critical median NLR value of 2.46, alluding to the fact preoperative NLR levers above this threshold correlate with deteriorating prognosis, showcased by reduced overall survival (OS) and progression-free survival (PFS).

Previously, several meta-analyses have been published on the association between NLR and the prognosis of patients with cervical cancer (10–12). In 2020, a meta-analysis evaluated the prognostic significance of the NLR in cervical cancer patients. This study demonstrated a notable association between NLR and survival outcomes in individuals with early-stage disease undergoing radical surgery (13). Nevertheless, numerous recent clinical studies have sought to further clarify the predictive value of NLR in cervical cancer, potentially contesting earlier conclusions (7, 14–19). Therefore, this updated meta-analysis aims to reexamine the prognostic significance of NLR across different stages of cervical cancer, including recurrent metastases, to provide the most current evidence-based understanding of NLR’s impact on disease prognosis.

## 2 Material and methods

### 2.1 Literature search

This study was conducted adherently to the Preferred Reporting Items for Systematic Reviews and Meta-Analyses (PRISMA2020) statement (20) and was registered in the International Prospective Register of Systematic Reviews (PROSPERO: CRD42024529817).

Researchers ZXY and FLJ independently developed the search strategy, formulating subject terms and keywords for database queries in PubMed, Embase, the Cochrane Library, and Web of Science, covering entries up to March 3, 2024. The search encompassed a diverse array of terms, including “Leukocytes, Polymorphonuclear,” “Polymorphonuclear Leukocytes,” “Neutrophil, Polymorphonuclear,” “Lymphoid Cells,” “Cells, Lymphoid,” “Cervical Neoplasm, Uterine,” “Neoplasm, Uterine Cervical Neoplasm,” “Cervical Neoplasms Uterine Cervical Cancer,” “Cervix Neoplasm,” “Cancer of the Uterine Cervix,” “Cancer of the Cervix,” “Cervical Cancer,” and “Cancer of Cervix.” The comprehensive search strategy is documented in **Supplementary Table 1**.

### 2.2 Study selection

Studies matching the following criterion were included: (1) Pathological diagnosis of cervical cancer in patients; (2) Exploration of pre- or post-treatment NLR’s prognostic impact on OS, PFS, disease-free survival (DFS) and recurrence. OS was defined as the time from postoperative day l to the time of death (excluding deaths due to non-tumor factors). PFS was defined as the time from postoperative day l to the time when the patient experienced tumor relapse/metastasis. DFS was defined as the time from the date of surgery to recurrence, death, or the last follow-up. Recurrence consisted of primary recurrence, distant metastasis, and primary recurrence plus distant metastasis. Time to recurrence was calculated from the date of cervical cancer diagnosis to the date of its recurrence. (3) Availability of hazard ratios (HR) with 95% confidence intervals (CI) or the ability to compute them; (4) Dividing patients into high and low NLR groups on the basis of predetermined breakpoints; (5) Full publication of studies; (6) Publication in English.

Exclusion criteria were: (1) Reviews, individual case reports, comments, conference abstracts, and letters; (2) Studies without sufficient data to compute HR and 95% CI; (3) Studies lacking survival data; (4) Studies with duplicated or overlapping data. Researchers ZXY and FLJ independently screened titles and abstracts, reviewed complete texts of studies for eligibilities, and settled disagreements through discussions.

### 2.3 Data extraction

Data extraction was executed independently by researchers ZXY and FLJ, with conflicts resolved by arriving at a consensus among all authors. Extracted data included the author’s name, publication year, geographic location of the study, research design, sample size, patient age, tumor stage, detection time, study duration, NLR cut-off points, follow-up period, and HR with 95% CI for OS and PFS. For studies reporting lymphocyte-neutrophil ratio (LNR) data, HR and 95% CI were converted to NLR by inverting values and swapping confidence limits to facilitate comparison.

### 2.4 Quality assessment

The quality of the studies included in our analysis was evaluated using the Newcastle-Ottawa Quality Assessment Scale (NOS), which appraises studies based on selection, comparability and disposition, with the highest achievable score being nine points (21). Studies obtaining scores in the range of 7 to 9 were considered of high quality.

### 2.5 Statistical analysis

The prognostic relevance of NLR in cervical cancer patients was assessed by pooling and 95% CIs, and LNR data was reformatted to NLR for consistency. Heterogeneity was evaluated using Cochran’s Q test and Higgins *I^2^* statistic (22), with the further utilization of a random effects model. To ascertain the robustness of results tied to OS and PFS, subgroup and sensitivity analyses were executed. Any probable publication bias was identified using funnel plots and Egger’s test, designating p-value of <0.05 as the threshold for statistical relevance. All statistical work was performed using STATA 15.0 and Review Manager 5.4.

## 3 Results

### 3.1 Study characteristics

**Figure 1:**
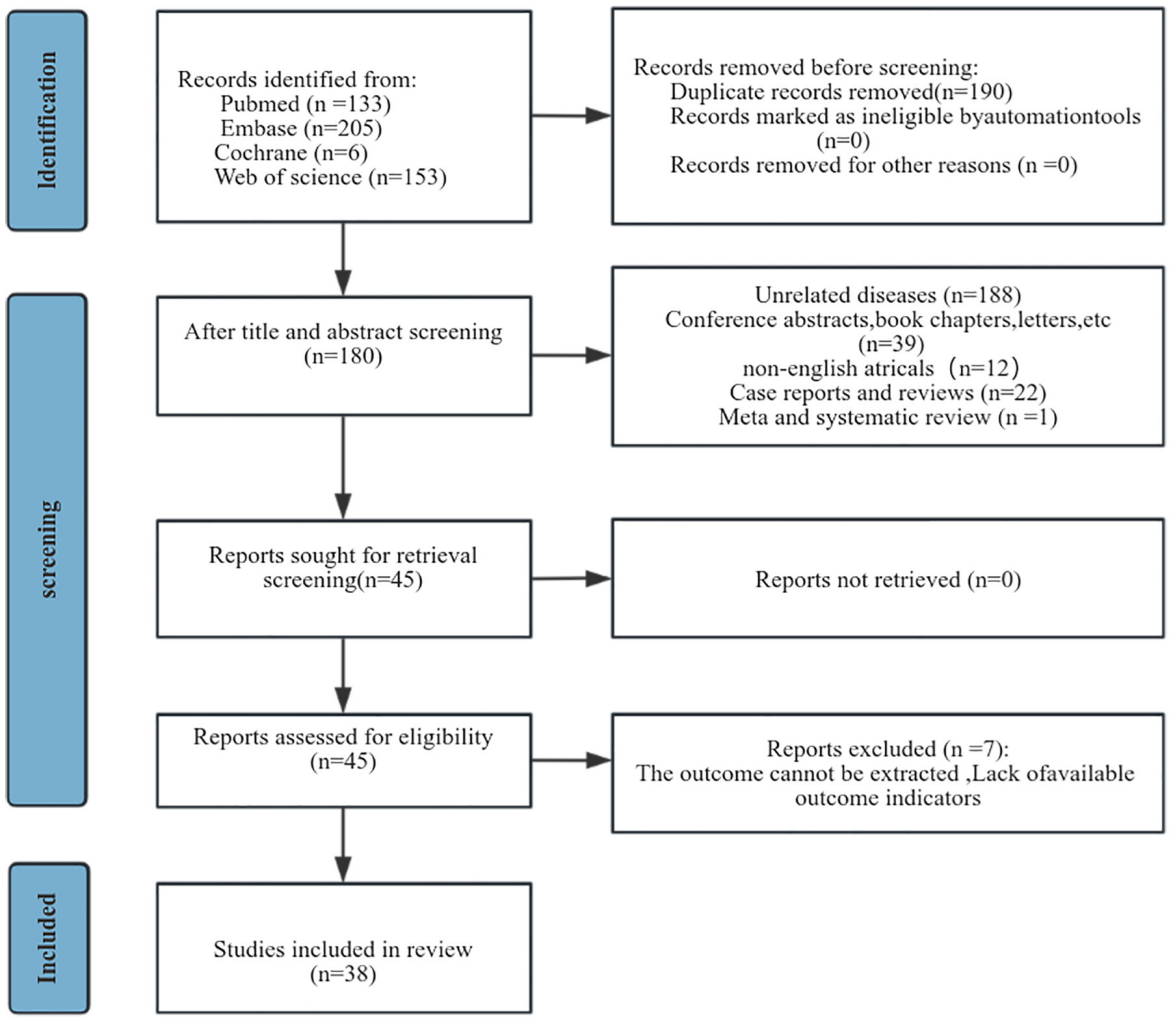
Flow chart of literature screening.

A preliminary search of the database yielded 497 articles. 190 articles were excluded due to the presence of duplicate publications. Following the title and abstract screening process, 262 studies were subsequently excluded. A full-text assessments was conducted on 45 studies, resulting in the exclusion of seven due to the absence of sufficient data pertinent to survival analyses (**Figure 1**). Finally, this meta-analysis encompassed 38 studies involving 10,246 patients (**Table 1**). Of these, 25 studies originated from Asian—predominantly China, Korea, and Japan—while the remainder were conducted in Europe and Americas. Each study was a retrospective cohort study, published in English, with publication dates ranging from 2014 to 2024. All studies segmented participants into high-NLR groups and low-NLR groups. Predominantly, NLR was measured prior to treatment. Regarding specific survival outcomes: 32 studies assessed the impact of NLR on OS, 18 on PFS, 2 on recurrence-free survival (RFS), 7 on DFS, 3 on recurrence, and 2 on distant metastases. The NLR threshold ranged from 1.6 to 6.91. The study populations consisted of individuals of all stages of cervical cancer, with four articles only on early stages and three only on advanced stages. Comprehensive details relevant to the 38 studies included in our analysis can be referenced in **Table 1**. Each study earned quality scores between 6 and 9 on the NOS, highlighting their high caliber (**Supplementary Table 2**).

**Table 1:** Baseline characteristics of include studies.

Author	study period	region	study design	Population	No. of patients	Mean Age	Mean follow-up	TNM stage	NLR threshold	Timing
Ayumi Taguchi 2021	2004-2015	Japan	Retrospective cohort	patients with recurrent cervical cancer after radiation-based therapy	89	67	16.4 months	I-IV	6.91	pre-treatment
Cem Onal MD 2016	2006-2014	Turkey	Retrospective cohort	patients given definitive ChRT for histologically proven cervical cancer	235	57	53months	I-IV	3.03	pre-treatment
Chunyu Liang 2022	2015-2019	China	Retrospective cohort	patients with biopsy-proven squamous cell carcinoma (SCC) of primary cervical cancer	78	55.1	32.5months	I-IV	3.87	pre-treatment
Federica Medici 2023 (17)	2007-2021	Italy	Retrospective cohort	Patients underwent definitive concurrent CRT, which involved a combination of external beam RT (EBRT) targeting the pelvic area	173	56	36months	I-IV	5	pre-treatment
Hamilton Trinh 2020	2008-2019	United States	Retrospective cohort	patients diagnosed with cervical cancer underwent definitive chemoradiotherapy (dCRT)	99	47	3.37years	I-IV	2.7	pre-treatment
Hong-Bing Wang 2023 (3)	2013-2015	China	Retrospective cohort	patients with cervical cancer who underwent RT were collected	178	53.85	NA	II-III	2.8	pre-treatment
HYUN JUNG LEE1 2020	2005-2016	Korea	Retrospective cohort	cervical cancer treat with CCRT	125	53.67	50 months	II-III	3.04	pre-treatment
Jenny Ling-Yu hen 2023	2016-2021	Taiwan	Retrospective cohort	patients with CC who received curative radiochemotherapy	138	60.1	33.8 months	I-IV	2.4	pre-treatment
Jeong Won Lee 2021	2008-2018	Korea	Retrospective cohort	clinicalandradiologicFIGO stageIB-IVAwithnootherevidenceof distantmetastasis	148	54.2	75 months	I-IV	2.34	pre-treatment
Ji-Hoon Sim2021	2006-2015	Korea	Retrospective cohort	patients who were diagnosed with cervical cancer underwent ORH or LRH	929	47.2	NA	I-IV	NA	pre-treatment
Joanna onska-Gmyrek 2018	2003-2008	Poland	Retrospective cohort	ervical cancer patients with FIGO stage IA–IV disease	94	53	66 months	I-IV	1.6	pre-treatment
Jun-Qiang Du 2023 (15)	2012-2017	China	Retrospective cohort	I–IIA cervical cancerhad undergone initial radical cervical cancer surgery	202	52.38	5years	I-II	3.75	pre-treatment
KEIICHIRO NAKAMURA 2016	2005-2014	Japan	Retrospective cohort	The clinicopathological characteristics of 32 patients with recurrThe primary treatment of these patients was CCRT	32	52.6	198days	IB-IIA	3.95	pre-treatment
KOHEI NAKAMURA 2018	1997-2013	Japan	Retrospective cohort	non-surgically treated patients with uterine cervical carcinoma	98	65	NA	I-IV	3.5	pre-treatment
Liang Chen MD 2016	2006-2009	China	Retrospective cohort	patients with FIGO stage Ib1–IIa cervical cancer, who underwent radical surgery	407	44	5years	IB1-IIA	2.42	pre-treatment
Luiz Claudio Santos Thuler 2021	2006-2009	Brazil	Retrospective cohort	women with CC, diagnosed and treated at a single referral cancer center	1266	49.8	4.51years	I-III	2.57	pre-treatment
Makito Mizunuma 2015	2005-2013	Japan	Retrospective cohort	patients who had stage IB1 to IV uterine cervical cancer underwent RT or CCRT	56	65.1	NA	I-IV	2.5	pre-treatment
Martina Ferioli 2023	2007-2021	Italy	Retrospective cohort	patients with locally advanced cervical cancer	173	56	36months	I-IV	3	pre-treatment
Matteo Bruno 2024 (8)	2012-2019	Italy	Retrospective cohort	patients with apparent early-stage cervical cancer who underwent primary surgery	174	47	53months	I-II	2.41	pre-treatment
Mengli Zhao 2023 (7)	2008-2018	China	Retrospective cohort	patients who receivedconcurrent chemoradiotherapy or radiotherapy	202	50	71 months	I-II	3.029	pre-treatment
Mingxia Cheng 2022	2019-2021	China	Retrospective cohort	patients with metastatic cervical cancer who underwent combination immunotherapy	70	51	NA	I-IV	5.33	pre-treatment
Myung-Hwa Jeong 2019	2001-2012	Korea	Retrospective cohort	patients with cervical cancer classified as International Federation of Gynecology and Obstetrics (FIGO) stage IIb to IVa who were treated using primary RT or CCRT	392	57	63.4months	IIB-IVA	2.8	pre-treatment
NAOYUKI IDA 2017	2004-2015	Japan	Retrospective cohort	patients whose cervical cancer recurred after undergoing concurrent chemoradiation therapy (CCRT), or radical hysterectomy with or without CCRT	79	52.4	NA	I-IV	2.9	pre-treatment
O.Abu-Shawer 2019	2006-2012	Jordan	Retrospective cohort	patients diagnosed with stage III or IV gynecological cancer, as confirmed by histopathology and/or radiology reports	72	56	NA	III-IV	4.1	pre-treatment
Oyeon Cho 2022	2001-2020	Korea	Retrospective cohort	cervical cancer patients treated with primary CRT after diagnosis	323	57	NA	I-IV	2.43	pre-treatment
Patrícia Santos Vaz de Lima 2020	2010-2018	Brazil	Retrospective cohort	patients with confirmed diagnosis of invasive cervical	102	51.72	NA	I-IV	4	pre-treatment
Pornprom Ittiamornlert 2018 (23)	2006-2017	Thailand	Retrospective cohort	cervical cancer patients with stage IVB disease,persistent disease, or recurrent disease who were treated by chemotherapy	355	51.9	5years	I-IV	3.6	pre-treatment
Sabyasachi Sarkar 2023 (18)	2017-2019	India	Retrospective cohort	patients treated with definitive chemoradiotherapy	208	50	NA	I-III	2.45	pre-treatment
Sevgi Ayhan 2022	2008-2018	Turkey	Retrospective cohort	patients who underwent radical hysterectomy for CC	163	49	NA	I-III	2.4	pre-treatment
Wei Chen 2021	2010-2020	China	Retrospective cohort	patients who had been diagnosed with LACC received NACT before surgery and had undergone radical hysterectomy	341	46.4	60.5months	IB2- IIA2	3.16	pre-treatment
Xia He 2018	2007-2009	China	Retrospective cohort	Patients with first diagnosed cervical cancer	229	44	83months	I-IV	1.6	pre-treatment
Xiang Fan 2023 (16)	2012-2017	China	Retrospective cohort	patients with cervical cancer who underwent surgical treatment and had pelvic lymph node metastasis	103	NA	63 months	IIIC1p	3.8	pre-treatment
Yan-Yang Wang 2016	2009-2010	China	Retrospective cohort	cervical cancer who underwent CCCRT	60	53	58 months	II-III	2	pre-treatment
Yong-Xia Li 2021 (5)	2011-2016	China	Retrospective cohort	patients diagnosed with stage IIb cervical cancer	260	51	NA	I-IV	2.49	pre-treatment
YOO-YOUNG LEE 2012	1996-2007	Korea	Retrospective cohort	Patients with clinically staged cervical carcinoma (IB toIVA)	1061	50	52.9 months	I-IV	1.9	pre-treatment
Youn Ji Kim 2019	2009-2016	Korea	Retrospective cohort	patients who received definitive chemoradiation for cervical cancer	107	55	39.9months	I-IV	2.33	pre-treatment
Yu Zhang MD 2014	2005-2008	China	Retrospective cohort	cervical carcinoma treated with radical hysterectomy and pelvic lymphadenectomy	460	44	69 months	I-II	2.213	pre-treatment
Zhenhua Zhang 2023 (19)	2007-2015	China	Retrospective cohort	patients with LASCC diagnosed for the first time and then treated with radical chemoradiotherapy	965	NA	NA	II-IV	2.91	pre-treatment

### 3.2 Meta-analysis results

#### 3.2.1 NLR and OS

**Figure 2:**
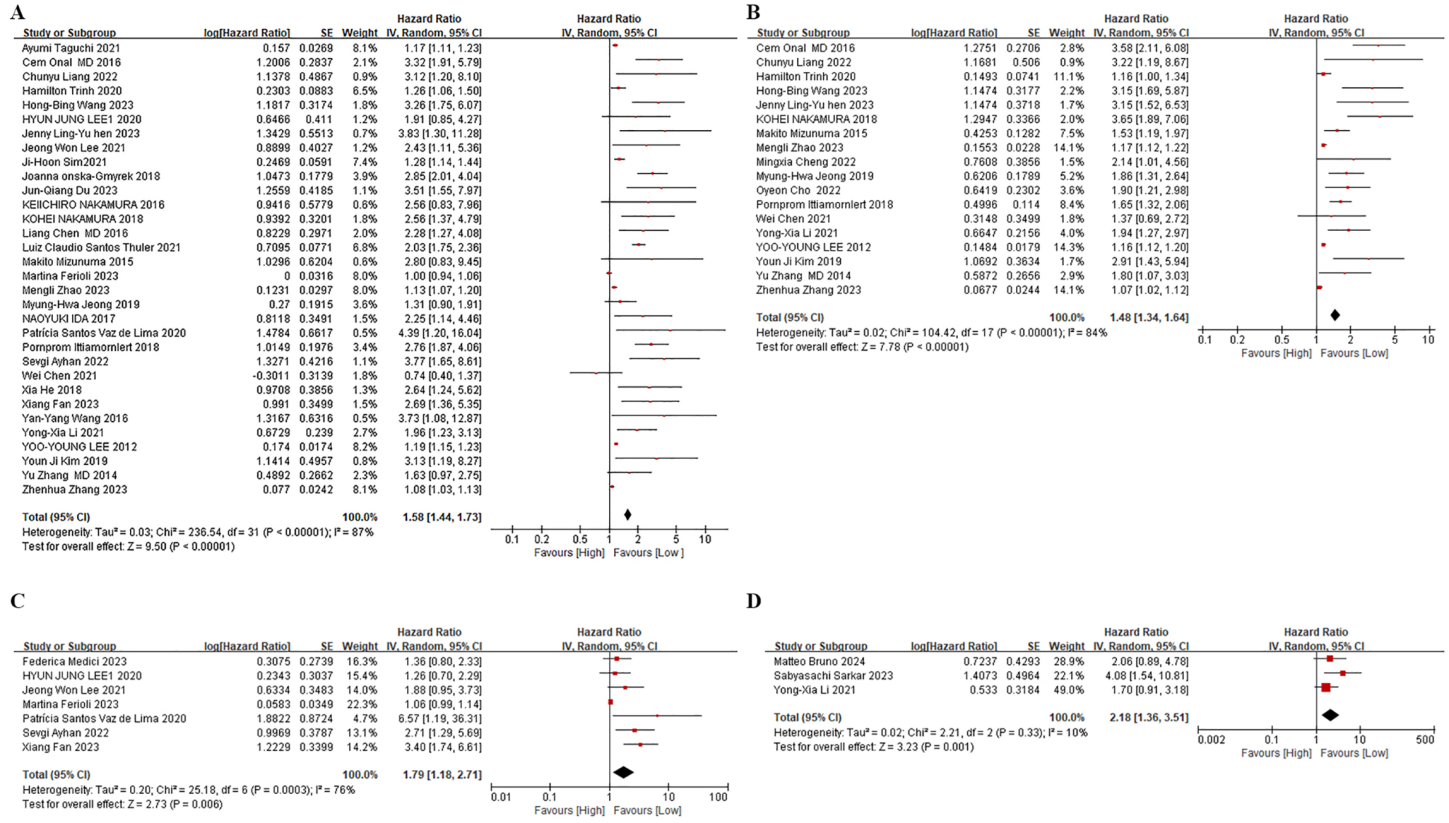
Forest plots. **(A)** Forest plots for the association between NLR and OS. **(B)** Forest plots for the association between NLR and PFS. **(C)** Forest plots for the association between NLR and DFS. **(D)** Forest plots for the association between NLR and Reccurence.

Among the 32 retrospective cohort studies examining the association between pre-treatment NLR and OS, significant heterogeneity was detected (*I^2^* = 87%, P < 0.00001), resulting in the adoption of a random-effects model (**Figure 2A**). Elevated pre-treatment NLR was notably associated with shorter OS (HR = 1.58, 95% CI: 1.44-1.74; P < 0.00001). Subgroup analyses, stratified by treatment regimen, patient age, study site, and NLR cut-off value, were presented in **Table 2**. Firstly, high NLR was linked to poorer OS in surgical-based (HR: 2.01; 95% CI: 1.61-2.52; P < 0.00001) and non-surgical treatments (radiotherapy and chemotherapy) (HR: 1.40; 95% CI: 1.25-1.56; P < 0.00001), with both statistical significance. Secondly, subgroup analyses based on patient age revealed that a high NLR was linked to shorter OS in individuals aged 50 years or older (HR: 1.62; 95% CI: 1.44-1.82; P < 0.00001) and in individuals younger than 50 years old (HR: 1.65; 95% CI: 1.35-2.02; P < 0.00001). Thirdly, subgroup analyses based upon study site showed that the effect of a high NLR on adverse OS was evident in patients from Asia (HR: 1.42; 95% CI: 1.30-1.56; P < 0.00001), Europe (HR: 2.36; 95% CI: 1.42-3.94; P = 0.0009) and the Americas (HR: 1.26; 95% CI: 1.06-1.50; P = 0.009). Furthermore, neither the low NLR threshold (HR: 1.69, 95% CI: 1.48-1.93; P < 0.00001) nor the high NLR threshold (HR 1.5, 95% CI: 1.29-1.75; P < 0.00001) influenced the prognostic impact of elevated NLR on adverse OS.

**Table 2:** Pooled HRs for OS and PFS in subgroup analyses.

Subgroup	OS				PFS			
	Study	HR [95%CI]	*P* value	*I* ^2^	Study	HR [95%CI]	*P* value	*I* ^2^
Total	32	1.58 [1.44-1.73]	<0.00001	87%	18	0.38 [0.16-0.90]	<0.00001	84%
Treatment								
Surgery-based combination therapy	12	2.01 [1.61-2.52]	<0.00001	89%	3	1.51 [1.02-2.24]	0.04	76%
non-surgical treatment	20	1.40 [1.25-1.56]	<0.00001	82%	15	1.63 [1.42-1.87]	<0.00001	85%
Mean/median age								
≥50y	22	1.62 [1.44-1.82]	<0.00001	85%	14	1.71 [1.50-1.96]	<0.00001	85%
<50y	8	1.65 [1.35-2.02]	<0.00001	87%	3	1.28 [1.00-1.63]	0.05	25%
Region								
Asia	25	1.42 [1.30-1.56]	<0.00001	78%	16	1.47 [1.32-1.63]	<0.00001	83%
Europe	6	2.36[1.42-3.94]	0.0009	96%	1	3.58 [2.11-6.08]	<0.00001	NA
America	1	1.26 [1.06-1.50]	0.009	NA	1	1.16 [1.00-1.34]	0.04	NA
NLR cut-off								
≥3	13	1.5 [1.29-1.75]	<0.00001	86%	7	2.02 [1.4-2.90]	0.0002	86%
<3	19	1.69 [1.48-1.93]	<0.00001	87%	11	1.42 [1.26-1.61]	<0.00001	83%

#### 3.2.2 NLR and PFS

Among the 18 studies that investigated pre-treatment NLR and PFS, a random-effects model was employed owing to considerable heterogeneity (*I^2^* = 84%, p < 0.00001) (**Figure 2B**). The combined findings indicate that an increased NLR was associated with shorter PFS in cervical cancer patients (HR: 1.48, 95% CI: 1.34-1.63; p < 0.00001, **Figure 2B**). In order to discern potential triggers for the heterogeneity, we carried out subgroup analyses, stratified by treatment regimen, patient age, study site, and cut-off value. First, in studies employing non-surgical treatments, such as radiotherapy and chemotherapy (HR: 1.63; 95% CI: 1.42-1.87; p < 0.00001), the PFS was inferior in the high NLR group. Conversely, no notable prognostic impact of NLR was observed in studies utilizing a blend of surgical and non-surgical treatments (HR: 1.51, 95% CI: 1.02-2.24; p = 0.04). Secondly, a subgroup analysis based on patient age demonstrated that high NLR significantly influenced PFS in studies involving patients aged 50 years or older. (HR: 1.71; 95% CI: 1.50-1.96; p < 0.00001). Nevertheless, no significant prognostic impact of NLR was observed in studies where patients were younger than 50 years old (HR: 1.28, 95% CI: 1.00-1.63; p = 0.05). Thirdly, based on the study location, subgroup analysis highlighted the significant prognostic impact of a high NLR on poorer PFS in Asia (HR: 1.47, 95% CI: 1.32-1.63; p < 0.00001), and Europe (HR: 3.58, 95% CI: 2.11-6.08; p < 0.00001). Conversely, in the Americas, no significant effect of high NLR on PFS was observed (HR: 1.16, 95% CI: 1.00-1.34; p = 0.04). In summary, NLR was not an effective predictor of PFS in patients receiving a combination of treatments based primarily on surgical therapy, in those aged less than 50 years, or in populations in the Americas. Heterogeneity analyses suggest that those aged less than 50 years may be a primary contributor to increased heterogeneity in this metric.

#### 3.2.3 NLR and other indicators

Data on pre-treatment NLR and DFS were extracted from seven studies. Owing to considerable heterogeneity among these studies (*I^2^ =* 76%, p = 0.006), we resorted to employing a random-effects model (**Figure 2C**). Consistent with our findings on OS and PFS, higher NLR levels were linked to reduced DFS in these patients (HR: 1.79, 95% CI: 1.18-2.71; p = 0.006, **Figure 2C**). Regarding recurrence, three studies indicated that elevated NLR correlated with a shorter interval to recurrence in these patients (HR: 2.18, 95% CI: 1.18-2.71; p = 0.006, **Figure 2D**). Moreover, two studies on RFS displayed a significant relationship between high NLR and shortened RFS (HR: 3.05, 95% CI: 1.79-5.19; p < 0.0001). Additionally, two studies assessing the impact of elevated NLR on distant metastases revealed a notable association (HR: 1.73, 95% CI: 1.20-2.50; P = 0.003), underscoring the broad prognostic relevance of NLR for various clinical outcomes in cervical cancer.

### 3.3 Sensitivity analysis

**Figure 3:**
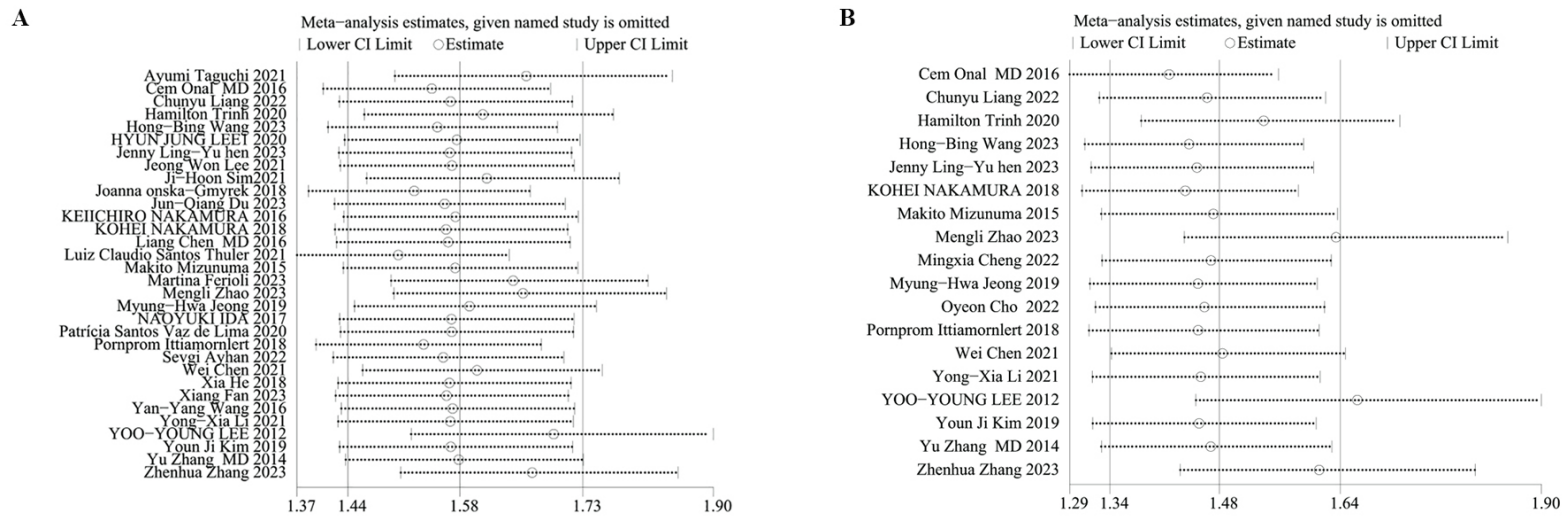
Sensitivity analysis of **(A)** OS and **(B)** PFS.

We performed sensitivity analyses on OS and PFS to evaluate the reliability of the results concerning the baseline NLR. Results displayed that the predictive worth of the NLR for OS and PFS persisted consistently even after sequentially excluding each study. This finding indicates that the overall impact upon OS (**Figure 3A**) and PFS (**Figure 3B**) outcomes were not singly dictated by any individual study, thereby confirming analysis robustness.

### 3.4 Publication bias

**Figure 4:**
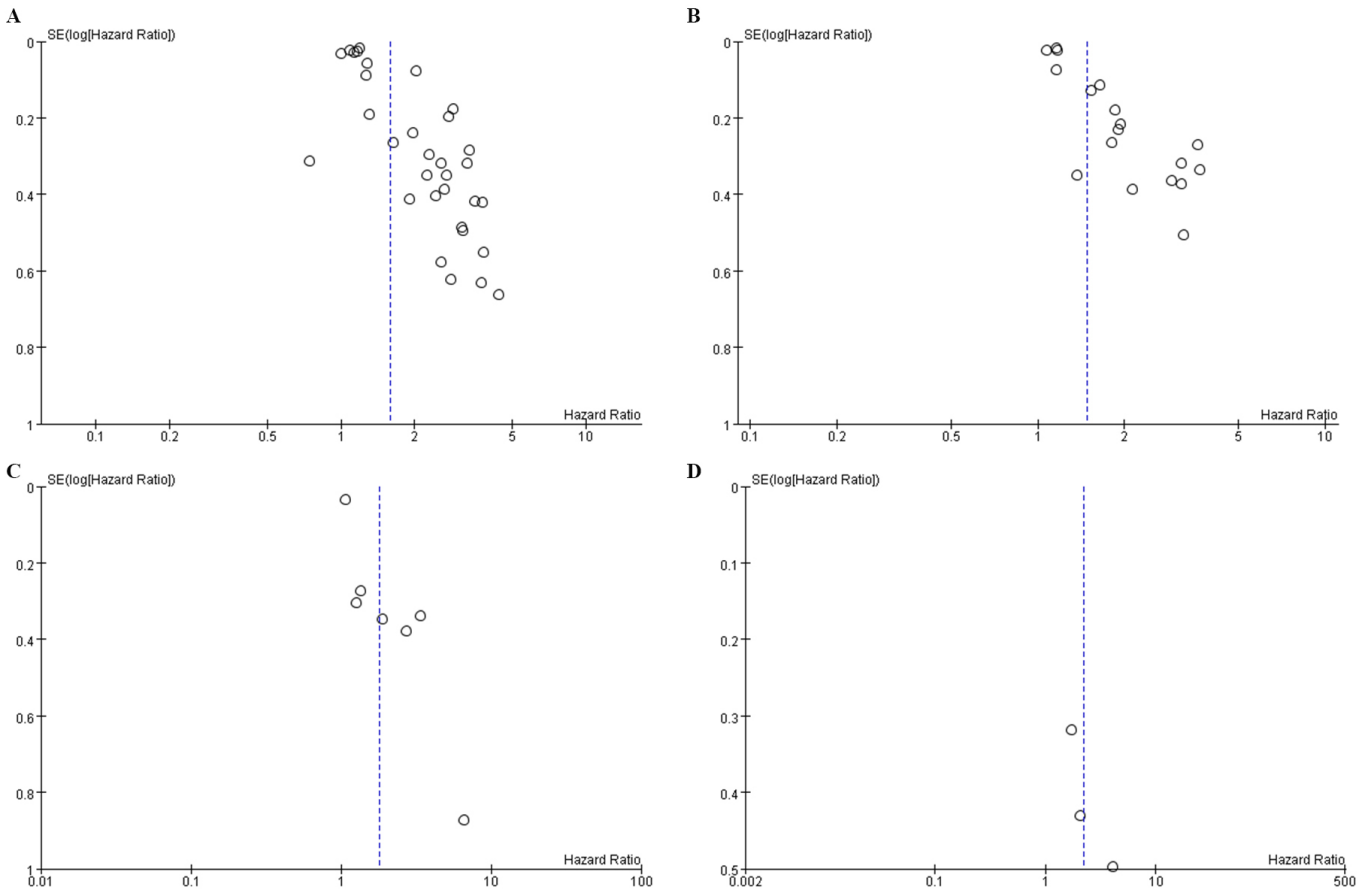
Funnel plot for the evaluation of publication bias for **(A)** OS **(B)** PFS **(C)** DFS and **(D)** Reccurence.

Publication bias was corroborated using a funnel plot and Egger’s test. Notably, the funnel plot for OS (**Figure 4A**) displayed asymmetry, indicating bias, substantiated by the Egger’s test (P = 0.0001). Similar inferences were made for PFS, evidenced by an asymmetric funnel plot (**Figure 4B**) and supported by the Egger’s test (P = 0.0001). The funnel plot for DFS (**Figure 4C**) and Reccurence (**Figure 4D**) did not displayed asymmetry.

## 4 Discussion

Recent studies have increasingly highlighted the link between elevated NLR and tumor progression and metastasis (24–27). The NLR stands as an indicator of the equilibrium struck between inflammation instigated by the tumor and immune responses opposing the tumor (28). A higher NLR might suggest an intensified pro-tumor inflammatory response coupled with a weakened in anti-tumor immune activity (7). Research has identified a correlation where elevated NLR values are linked to reduced survival rates in cervical cancer patients (16). Lima et al. (29) reported that a high NLR serves as an independent determinant of both DFS and OS, positing it as a prognostic marker for poorer outcomes in invasive cervical tumors. Similarly, Ittiamornlert et al. (23) observed that in patients undergoing chemotherapy for stage IVB, persistent, or recurrent cervical cancer, an NLR ≥ 3.6 independently predicted adverse tumor outcomes, affecting overall response rate, PFS and OS. Our analysis corroborates these findings, showing that high NLR significantly impacts both poorer OS and PFS adversely, with a notably stronger prognostic significance for OS. This relationship persists across various treatment regimens, age groups, study locations, and irrespective of NLR thresholds, aligning closely with previous meta-analytical data (10). In addition, our review included seven studies addressing the impact of high NLR on DFS, three on recurrence, two on RFS, and two on distant metastases. These studies confirmed significant associations of high NLR with decreased DFS (HR: 1.79, 95% CI: 1.18-2.71; P = 0.006), increased recurrence rates (HR: 2.18, 95% CI: 1.36-3.51; P = 0.001), shorter RFS (HR: 3.05, 95% CI: 1.79-5.19; P < 0.0001), and higher risks of distant metastases (HR: 1.73, 95% CI: 1.20-2.50; P = 0.003).

Subgroup analyses pertaining to the stages of cervical cancer were not conducted owing to a couple of key considerations. Many studies presented combined data for patients across stages I-IV. Given that the initial treatment is primarily determined by the stage of cancer, these datasets invariably included a blend of surgical-based interventions. Certain studies solely examined patients with locally advanced conditions and recurrent metastases, predominantly employing radiotherapy-based treatment regimens (30, 31). Consequently, we executed a subgroup analysis for these two specific treatment modalities. Interestingly, the impact of high NLR on OS was significantly pronounced for both surgical and non-surgical interventions. However, when it comes to PFS, a high NLR didn’t display substantial predictive value for the group undergoing surgical treatment. This could be potentially attributed to the fact that patients in the surgical group were generally early-stage tumor patients and thus, had a relatively lower risk of tumor progression and recurrence following surgery. Besides, high NLR had no predictive value for patients younger than 50 years, suggesting that a younger age could serve as a protective factor against prolonged PFS, with an *I^2^ =* 25% indicating that this younger demographic contributed to increased heterogeneity.

Prior research has underscored the critical role of inflammation in tumor initiation, growth, invasion, and metastasis (32). Inflammatory cells, particularly lymphocytes and neutrophils, play a pivotal role in these processes. For instance, neutrophils support immune reactions via discharging cytokines, antigens, and chemokines, generating inflammatory mediators that foster a tumor-friendly microenvironment, aiding in tumor angiogenesis through metalloproteinases, inducing tumor suppressor gene mutations, and potentially reducing immune defense against tumors by decreasing lymphocytes. Additionally, even in the early stages of cancer, circulating tumor cells may be present, which are often associated with neutrophil clusters (15).

This meta-analysis, consolidating data from 10,246 patients, appraised the prognostic worth of NLR at varying stages of cervical cancer, including cases with recurrent metastases. A sizeable positive link was discovered between NLR prior to treatment and both OS and PFS. Future research efforts should delve into the relationship between NLR post-treatment and the patient’s prognosis. To investigate the connection between high NLR and OS and PFS, we initiated a subgroup assessment based on differing treatments. Our conclusions were deemed robust after a sensitivity analysis. Nevertheless, Egger’s test indicated a potential publication bias, likely stemming from the predominance of data from Asian countries. This geographical concentration necessitates caution when generalizing these findings to non-Asian populations. Further research is needed to confirm NLR’s prognostic value in non-Asian cervical cancer patients. Moreover, the retrospective nature of the included studies may introduce confounding factors affecting the reliability of the results. In addition, the varying NLR thresholds (1.6 to 6.9) among studies bring about the heterogeneity of the meta-analysis. Establishing a standardized NLR threshold is crucial for future research reliability and comparability.

## 5 Conclusion

Higher pre-treatment NLR values are considerably linked with OS, PFS, DFS and recurrence in cervical cancer patients, underscoring potential prognostic relevance of NLR. However, the heterogeneity and publication bias presented in the included studies necessitate further comprehensive, perspective research to establish the prognostic reliability of NLR in cervical cancer.

## Data Availability

The original contributions presented in the study are included in the article/**Supplementary Material**. Further inquiries can be directed to the corresponding author.
