# Supplementary material for: Evaluating the prognostic relevance of neutrophil-to-lymphocyte ratio in cervical cancer: a systematic review and meta-analysis
Source: Front Oncol. 2024 Dec 23;14:1461175. doi: 10.3389/fonc.2024.1461175 (PMC11701371; doi:10.3389/fonc.2024.1461175)
Supplement: Supplementary file 1 [file DataSheet1.docx]

**Supplementary Table S1** Literature search process 20240303

Predictive value of neutrophil to lymphocyte ratio for the prognosis of cervical cancer : a systematic review and meta-analysis

Neutrophil

Leukocytes, Polymorphonuclear

Leukocyte, Polymorphonuclear

Polymorphonuclear Leukocyte

Polymorphonuclear Leukocytes

Polymorphonuclear Neutrophils

Neutrophil, Polymorphonuclear

Polymorphonuclear Neutrophil

LE Cells

Cell, LE

LE Cell

Neutrophil Band Cells

Band Cell, Neutrophil

Neutrophil Band Cell

Lymphocyte

Lymphoid Cells

Cell, Lymphoid

Cells, Lymphoid

Lymphoid Cell

Cervical Neoplasm, Uterine

Neoplasm, Uterine Cervical

Uterine Cervical Neoplasm

Neoplasms, Cervical

Cervical Neoplasms

Cervical Neoplasm

Neoplasms, Cervix

Cervix Neoplasm

Neoplasm, Cervix

Cervix Neoplasms

Cancer of the Uterine Cervix

Cancer of the Cervix

Cervical Cancer

Cancer, Cervical

Cervical Cancers

Uterine Cervical Cancer

Cancer, Uterine Cervical

Cervical Cancer, Uterine

Uterine Cervical Cancers

Cancer of Cervix

Cervix Cancer

Cancer, Cervix

PUBMED-133

(((("Neutrophils"[Mesh]) OR ((((((((((((((Neutrophil) OR (Leukocytes, Polymorphonuclear)) OR (Leukocyte, Polymorphonuclear)) OR (Polymorphonuclear Leukocyte)) OR (Polymorphonuclear Leukocytes)) OR (Polymorphonuclear Neutrophils)) OR (Neutrophil, Polymorphonuclear)) OR (Polymorphonuclear Neutrophil)) OR (LE Cells)) OR (Cell, LE)) OR (LE Cell)) OR (Neutrophil Band Cells)) OR (Band Cell, Neutrophil)) OR (Neutrophil Band Cell))) AND (("Lymphocytes"[Mesh]) OR (((((Lymphocyte) OR (Lymphoid Cells)) OR (Cell, Lymphoid)) OR (Cells, Lymphoid)) OR (Lymphoid Cell)))) AND (ratio)) AND (("Uterine Cervical Neoplasms"[Mesh]) OR ((((((((((((((((((((((Cervical Neoplasm, Uterine) OR (Neoplasm, Uterine Cervical)) OR (Uterine Cervical Neoplasm)) OR (Neoplasms, Cervical)) OR (Cervical Neoplasms)) OR (Cervical Neoplasm)) OR (Neoplasms, Cervix)) OR (Cervix Neoplasm)) OR (Neoplasm, Cervix)) OR (Cervix Neoplasms)) OR (Cancer of the Uterine Cervix)) OR (Cancer of the Cervix)) OR (Cervical Cancer)) OR (Cancer, Cervical)) OR (Cervical Cancers)) OR (Uterine Cervical Cancer)) OR (Cancer, Uterine Cervical)) OR (Cervical Cancer, Uterine)) OR (Uterine Cervical Cancers)) OR (Cancer of Cervix)) OR (Cervix Cancer)) OR (Cancer, Cervix)))

Embase-205


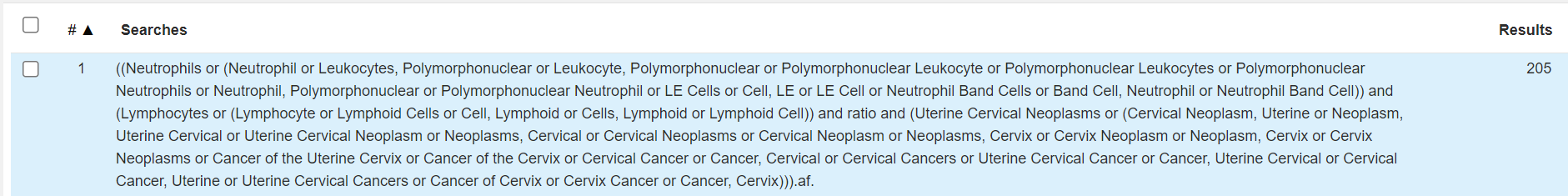


Cochrane-6
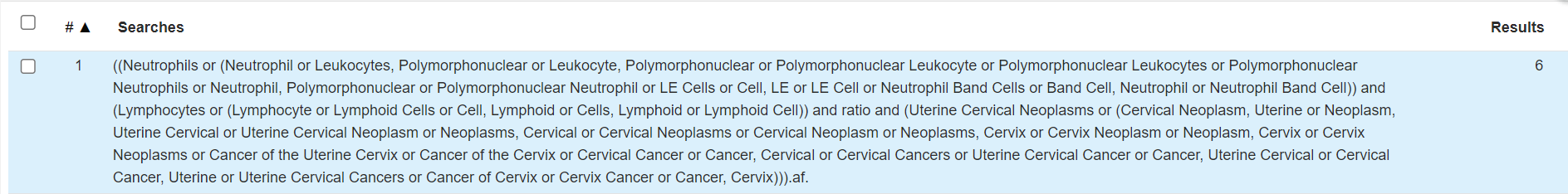


WOS-153

((((Neutrophils) OR ((((((((((((((Neutrophil) OR (Leukocytes, Polymorphonuclear)) OR (Leukocyte, Polymorphonuclear)) OR (Polymorphonuclear Leukocyte)) OR (Polymorphonuclear Leukocytes)) OR (Polymorphonuclear Neutrophils)) OR (Neutrophil, Polymorphonuclear)) OR (Polymorphonuclear Neutrophil)) OR (LE Cells)) OR (Cell, LE)) OR (LE Cell)) OR (Neutrophil Band Cells)) OR (Band Cell, Neutrophil)) OR (Neutrophil Band Cell))) AND ((Lymphocytes) OR (((((Lymphocyte) OR (Lymphoid Cells)) OR (Cell, Lymphoid)) OR (Cells, Lymphoid)) OR (Lymphoid Cell)))) AND (ratio)) AND ((Uterine Cervical Neoplasms) OR ((((((((((((((((((((((Cervical Neoplasm, Uterine) OR (Neoplasm, Uterine Cervical)) OR (Uterine Cervical Neoplasm)) OR (Neoplasms, Cervical)) OR (Cervical Neoplasms)) OR (Cervical Neoplasm)) OR (Neoplasms, Cervix)) OR (Cervix Neoplasm)) OR (Neoplasm, Cervix)) OR (Cervix Neoplasms)) OR (Cancer of the Uterine Cervix)) OR (Cancer of the Cervix)) OR (Cervical Cancer)) OR (Cancer, Cervical)) OR (Cervical Cancers)) OR (Uterine Cervical Cancer)) OR (Cancer, Uterine Cervical)) OR (Cervical Cancer, Uterine)) OR (Uterine Cervical Cancers)) OR (Cancer of Cervix)) OR (Cervix Cancer)) OR (Cancer, Cervix))) (Topic)
