# Supplementary material for: Evaluating the prognostic relevance of neutrophil-to-lymphocyte ratio in cervical cancer: a systematic review and meta-analysis
Source: Front Oncol. 2024 Dec 23;14:1461175. doi: 10.3389/fonc.2024.1461175 (PMC11701371; doi:10.3389/fonc.2024.1461175)
Supplement: Supplementary file 2 [file Table1.docx]

| **Supplementary Table S2** Quality evaluation of the eligible studies with Newcastle–Ottawa scale | | | | | | | | | |
| --- | --- | --- | --- | --- | --- | --- | --- | --- | --- |
| Study | Selection | | | | Comparability | | Outcome | | |
|  | Representative-ness | Selection of  non-exposed | Ascertainment  of exposure | Outcome not present at start | Comparability on most important factors | Comparability on other risk factors | Assessment of outcome | Long enough follow-up (median≥1 year) | Adequacy  (completeness) of follow-up |
| Xingping Han，2023 |  |  |  |  |  |  |  |  |  |
| Jun-Qiang Du，2023 | * | * | * | * | * | * | * | * | * |
| Ayumi Taguchi,  2021 | * | * | * | * | * | * | * | * | * |
| Corinne A. Calo,  2022 | * | * | * | * | * | * | * | * | * |
| Patrícia Santos Vaz de Lima,2020 | * | * | * | * | - | - | * | * | * |
| Pornprom Ittiamornlert  2018 | * | * | * | * | * | - | * | * | * |
| Yong-Xia Li,2021 | * | * | * | * | - | * | * | * | * |
| Liang Chen, MD  2016 | * | * | * | * | - | * | * | * | * |
| Mengli Zhao  ，2023 | * | * | * | * | * | - | * | * | * |
| Yu Zhang, MD  2014 | * | * | * | * | - | * | * | * | * |
| Joanna onska-Gmyrek，2018 | * | * | * | * | - | - | * | * | * |
| MeiLin Zhu,2018 | * | * | * | * | - | * | * | * | * |
| YOO-YOUNG LEE,2012 | * | * | * | * | * | - | * | * | * |
| Jeong Won Lee,2021 | * | * | * | * | - | * | * | * | * |
| KEIICHIRO NAKAMURA,2016 | * | * | * | * | - | * | * | * | * |
| Xiang Fan，2023 | * | * | * | * | - | * | * | * | * |
| Jenny Ling-Yu hen,2023 | * | * | * | * | - | * | * | * | * |
| KOHEI NAKAMURA,2018 | * | * | * | * | - | * | * | * | * |
| Cem Onal, MD，2016 | * | * | * | * | - | * | * | * | * |
| Hamilton Trinh，2020 | * | * | * | * | - | * | * | * | * |
| HYUN JUNG LEE1，2020 | * | * | * | * | - | * | * | * | * |
| Yan-Yang Wang,2016 | * | * | * | * | - | * | * | * | * |
| Sevgi Ayhan，2022 | * | * | * | * | - | * | * | * | * |
| Matteo Bruno 2024 | * | * | * | * | - | * | * | * | * |
| Makito Mizunuma  2015 | * | * | * | * | - | * | * | * | * |
| Luiz Claudio Santos Thuler，2021 | * | * | * | * | - | * | * | * | * |
| Zhenhua Zhang,2023 | * | - | * | * | - | * | - | * | * |
| Martina Ferioli,2023 | * | - | - | * | - | * | * | * | * |
| Chunyu Liang  2022 | * | * | * | * | - | * | * | * | * |
| Federica Medici  ，2023 | * | * | * | * | - | * | * | * | * |
| Wei Chen，2021 | * | * | * | * | - | * | - | * | * |
| Ji-Hoon Sim  2021 | * | * | * | * | - | * | * | * | * |
| Oyeon Cho ,2022 | * | * | * | * | - | * | * | * | * |
| O.Abu-Shawer  ,2019 | * | * | * | * | - | * | * | * | * |
| R.E. Fullerton,  2023 | * | * | * | * | - | * | * | * | * |
| S. Garcia,2016 | * | * | * | * | - | * | * | * | * |
| Youn Ji Kim,2019 | * | * | * | * | - | * | * | * | * |
| Meilian,2022 | * | * | * | * | - | * | * | * | * |
| WANG Dan,2013 | * | * | * | * | - | * | * | * | * |
| I Putu Yuda Prabawa,2019 | * | * | * | * | - | * | - | - | * |
| Mingxia Cheng,  2022 | * | * | * | * | - | * | * | * | * |
| Sabyasachi Sarkar  ,2023 | * | * | * | * | - | * | * | * | * |
| NAOYUKI IDA,2017 | * | * | * | * | - | * | * | * | * |
| Myung-Hwa Jeong,2019 | * | * | * | * | - | * | * | * | * |
| Xia He  ,2018 | * | * | * | * | - | * | * | * | * |
| Hong-Bing Wang  ,2023 | * | * | * | * | - | * | * | * | * |
| *indicates criterion met; - indicates significant of criterion not met. | | | | | | | | | |
